# Direct and sensitive detection of sulfide ions based on one-step synthesis of ionic liquid functionalized fluorescent carbon nanoribbons†

**DOI:** 10.1039/c9ra07701d

**Published:** 2019-11-15

**Authors:** Tao Luo, Xiaobo Wang, Yuting Qian, Junjie Liu, Lequn Li, Jiyang Liu, Jie Chen

**Affiliations:** Affiliated Tumor Hospital of Guangxi Medical University 71 Hedi Road Nanning 530021 PR China jiechen185@163.com; Department of Chemistry, Zhejiang Sci-Tech University 928 Second Avenue, Xiasha Higher Education Zone Hangzhou 310018 PR China liujy@zstu.edu.cn

## Abstract

Despite widely reported fluorescence sensors for cations, direct detection of anions is nevertheless still rare. In this work, ionic liquid-functionalized fluorescent carbon nanoribbons (IL-CNRs) are one-step synthesized and serve as the fluorescent probes for direct and sensitive detection of sulfide ions (S^2−^). The IL-CNRs are synthesized based on electrochemical exfoliation of graphite rods in a water-IL biphasic system. The as-prepared IL-CNRs exhibit uniform structure, high crystallinity, strong blue fluorescence (absolute photoluminescence quantum yield of 11.4%), and unique selectivity towards S^2−^. Based on the fluorescence quenching of IL-CNRs by S^2−^, a fluorescence sensor is developed for direct, rapid and sensitive detection of S^2−^ in the range of 100 nM to 1 μM and 1–300 μM with a low detection limit (LOD, 85 nM). Moreover, detection of S^2−^ in a real sample (tap water) is also demonstrated.

## Abstract 2

Sensitive detection of sulfide ions is realized based on one-step synthesis of ionic liquid functionalized fluorescent carbon nanoribbons.

## Graphical abstract

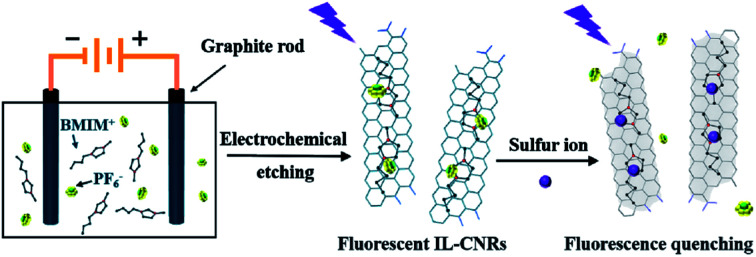

## 1. Introduction

Inorganic anions play a crucial role in industrial, biological and environmental fields.^1^ For instance, the sulfide (S^2−^) ion is highly toxic and harmful to human health and the ecological environment.^2–4^ Usually, the S^2−^ ion is frequently distributed in natural water and wastewater samples because it is widely used in industrial processes (*e.g.* sulfur and sulfuric acid production, petroleum refineries, paper and pulp manufacturing, and sewage processing industries) and is also produced in biological systems (*e.g. via* reduction of sulfate ions by microbes or anaerobic processes of S-containing enzymes).^4–6^ As the S^2−^ ion can stimulate the mucosa and has an extreme effect on the nervous system, exposure to S^2−^ ions leads to unconsciousness and respiratory paralysis and is closely related to various diseases including Alzheimer's disease, Down's syndrome and cirrhosis of the liver.^7–10^ Therefore, simple, rapid and sensitive detection of S^2−^ is of great importance.

In comparison with other technologies including gas chromatography, ion chromatography, titrimetry, colorimetry, and electrochemical method, fluorescent sensors are particularly attractive because of high sensitivity, simple operation and rapid detection.^3,11–17^ Recently, fluorescent sensors based on different fluorescent probes have been widely reported for detection of cations.^18,19^ However, only few researches focus on indirect detection of anions using fluorescent off–on strategy. For instance, Na *et al.* reported that Cu^2+^ ions caused aggregation of the GQDs and thereby quenched fluorescence.^12^ As the GQDs-Cu^2+^ aggregates can be dissociated by adding S^2−^ ions and result in fluorescence turn-on, fluorescent detection of S^2−^ ions could be realized. Based on the same off–on strategies, graphitic carbon nitride quantum dots-Hg^2+^,^7^ nitrogen and sulfur co-doped carbon dots (CDs)-Hg^2+^,^6^ CDs-gold nanoparticles,^10^ silver nanoparticles capped with CDs,^9^ CDs-MnO_2_ nanosheets,^13^ quinoline-Zn^2+^,^2^ calix[4]arene-Cu^2+^,^11^ and organic semiconductor polymer nanodots-Cu^2+^ systems^8^ were also applied for the detection of S^2−^ ions. However, it still remains great challenge for improving the chemical selectivity of fluorescent probe to realize the direct detection of S^2−^.

Carbon-based fluorescent nanomaterials have attracted considerable interests as probes in fluorescent sensing due to good solubility, highly tunable photoluminescence properties and biocompatibility.^20–24^ Carbon nanoribbons (CNRs), which is thin elongated strips of sp^2^ bonded carbon atoms, have emerged as an exciting one-dimensional nanomaterial because of their large length-to-width ratio, abundant edge sides, high effective surface area, good flexibility, high conductivity, and tunable fluorescence.^25–28^ Due to the large interfacial area for π–π stacking in basal planes, CNRs exhibit potentials for the synthesis of advanced functional materials through simple modification or functionalization.

Ionic liquids (ILs) have been widely used in many fields because of a series of excellent physical and chemical properties.^21,28,29^ Particularly, ILs can easily combine with carbon nanomaterials due to π–π or cation–π interactions and the obtained composite materials exhibit unique characteristics.^21,28–31^ For instance, Wang *et al.* prepared highly charged CDs through one-pot pyrolysis with citric acid as carbon source and ionic liquid (1-aminopropyl-3-methylimidazolium bromide, [APMIm][Br]) as capping agent.^32^ Those IL-modified CDs exhibit capability of anion exchange and are able to act as fluorescent probe for direct detection of Fe(CN)_6_^3−^ anion. Our group also prepared IL-GQDs nanomaterial through post-modification of hydroxyl-functionalized GQDs (OH-GQDs) with 1-butyl-3-methyl imidazolium tetrafluoroborate (BMIMBF_4_) under ultrasonic treatment. The as-prepared IL-GQDs composite also displayed sensitive response towards Fe(CN)_6_^3−^, owing to the anion exchange ability of IL.^33^ Inspired by these evidences, IL modified fluorescent nanocarbon materials possess great potentials for the direct detection of anions.

In this work, in contrast to anion detection based on fluorescence off–on mode, a new type of ionic liquid-functionalized fluorescent carbon nanoribbons (IL-CNRs) were prepared using one-step electrochemical method, which is able to achieve direct and sensitive detection of sulfide ions. The IL-CNRs were *in situ* synthesized based on electrochemical exfoliation of graphite rods in water-IL (1-butyl-3-methylimidazolium hexafluorophosphate, [BMIM]PF_6_) biphasic system. The obtained IL-CNRs exhibit bright fluorescence, high crystallinity and uniform structure. Based on the selective fluorescent quenching by S^2−^, IL-CNRs can be served as fluorescent probes for direct and sensitive detection of S^2−^. In comparison with the previous cation-based fluorescent sensor, we for the first time demonstrate the application of IL-CNRs for direct and selective detection of S^2−^.

## 2. Experimental

### 2.1 Materials and reagents

High-purity graphite rods were purchased from China National Medicines Co. Ltd (China). 1-Butyl-3-methylimidazolium hexafluorophosphate ([BMIM]PF_6_) (>99%), ascorbic acid (AA), and 4-(2-hydroxyethyl)-1-piperazineethanesulfonic acid (HEPES), Na_2_S were purchased from Sigma Aldrich. Aqueous solutions of Na^+^, K^+^, Zn^2+^, Mg^2+^, Fe^3+^, Ca^2+^, Al^3+^, Ni^2+^, Cu^2+^, Cd^2+^ were prepared from their chloride salts. Solutions of Pb^2+^, Ag^+^ and Hg^2+^ were prepared from their nitrate salts. Aqueous solutions of CH_3_COO^−^, HCO_3_^−^, H_2_PO_4_^−^, HPO_4_^2−^, NO_2_^−^, S^2−^, S_2_O_3_^2−^ were prepared from their sodium salts. SCN^−^, Cl^−^, NO_3_^−^, Br^−^ were prepared from their potassium salts. All chemicals were of analytical grade and used without further treatment. Aqueous solutions were prepared with ultrapure water (18.2 MΩ cm, Milli-Q, Millipore).

### 2.2 Characterizations

Transmission electron microscopy (TEM) micrographs were obtained using a JEM-2100F transmission electron microscope (JEOL Ltd, Japan) at accelerating voltage of 200 kV. X-ray photoelectron spectroscopy (XPS) measurements were carried out on a PHI5300 at operating voltage of 14 kV (PE Ltd., USA). The Fourier transform infrared spectra (FTIR) were conducted with a Nicolet 5700 FTIR spectrometer (Thermo Electron Scientific Instruments Corp., USA). Fluorescence spectra were measured using FL 3C-11 spectrofluorometer (Horiba Scientific, USA).

### 2.3 One-step electrochemical synthesis of IL-CNRs

The IL-CNRs were one-step synthesized by electrochemical exfoliation of graphite rods. A two electrodes system was adopted with graphite rods (*d* = 3 mm) as both anode and cathode. The electrolyte was water-IL ([BMIm][PF_6_]) biphasic system (V/V, 4 : 6). Graphite rods were vertically inserted into electrolyte solution at a distance of 3 cm.^21^ A static voltage of 8 V was applied on graphite rod for 4 h with stirring the electrolyte. Afterwards, the resultant solution was centrifuged at 12 000 rpm for 10 min to remove large particles. To acquire IL-CNRs, the supernatant solution was then filtered using a 0.22 μm membrane and dialyzed in a dialysis bag (with cut-off molecular weight of 1000 Da), which is followed by freeze-drying.

### 2.4 Fluorescent detection of S^2−^

The selectivity of IL-CNRs towards different anions and cations were evaluated by measuring the fluorescent intensity of IL-CNRs solution in presence of different cations (Na^+^, K^+^, Zn^2+^, Mg^2+^, Fe^3+^, Ca^2+^, Al^3+^, Ni^2+^, Cu^2+^, Cd^2+^) or anions (CH_3_COO^−^, HCO_3_^−^, H_2_PO_4_^−^, SCN^−^, HPO_4_^2−^, S^2−^, Cl^−^, NO_3_^−^, NO_2_^−^, Br^−^, S_2_O_3_^2−^).

For S^2−^ detection, standard stock solutions of S^2−^ (0.1 M) were prepared and further diluted stepwise to obtain different concentrations. HEPES buffer (0.1 M, pH 7.0) containing AA (25 μM) was applied as the buffer solution. After reaction of IL-CNRs with different amount of S^2−^ for 1 min at room temperature, the fluorescent intensity of the solution was recorded with the excitation wavelength fixed at 340 nm.

The relative fluorescence ratio (*F*/*F*_0_) or relative fluorescence quenching ratio (*F*_0_ − *F*/*F*_0_) were used to evaluate the quenching of IL-CNRs caused by ion, where *F*_0_ and *F* represent the fluorescent intensity of IL-CNRs solution in the absence and presence of ion, respectively.

## 3. Results and discussion

### 3.1 Preparation and structure characterizations of IL-CNRs

**Fig. 1:**
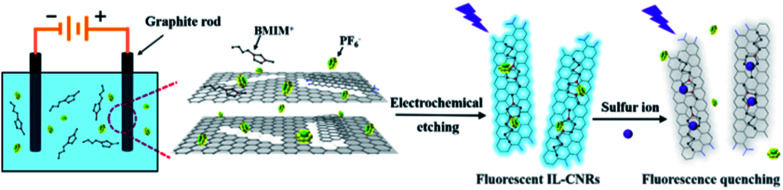
Schematic illustration for the preparation of IL-CNRs and detection of sulfide ions.

**Fig. 2:**
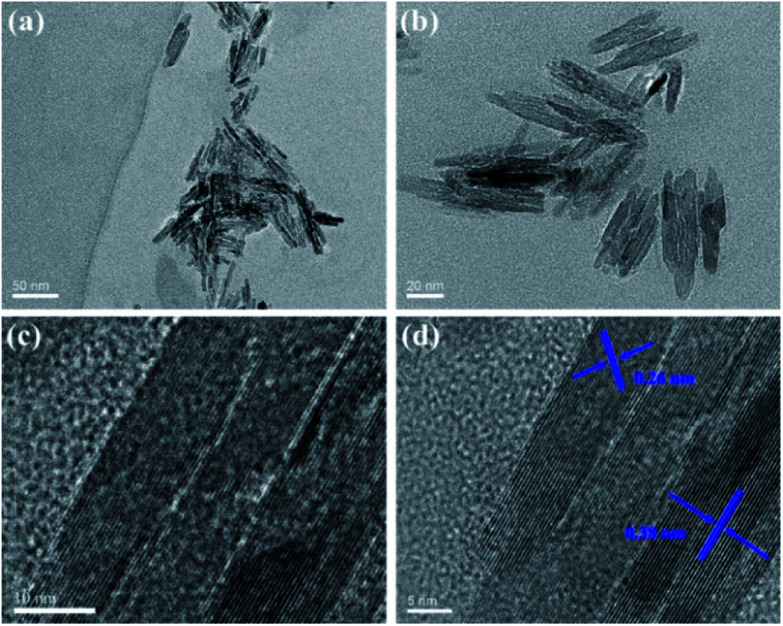
TEM (a–c) and HRTEM (d) images of IL-CNRs at different magnification. The indicated data are lattice parameters of IL-CNRs.

Electrochemical synthesis has particularly attracted attention owning to the inexpensive apparatus and easy operation under ambient conditions.^34–36^ As illustrated in Fig. 1, ionic liquid-functionalized fluorescent carbon nanoribbons (IL-CNRs) were easily synthesized through one-step electrochemical exfoliation of graphite rods in water-IL (1-butyl-3-methylimidazolium hexafluorophosphate, [BMIM]PF_6_) biphasic system. Owing to high ionic conductivity and wide electrochemical potential window, ILs shows potential as electrolyte in electrochemical synthesis.^17,24^ In the process of electrochemical exfoliation, the colour of electrolyte turned from colourless to pale yellow and afterward turned dark brown due to rapid flaking-off of abundant IL-CNRs. In comparison with the same procedure in pure water, this phenomenon cannot be observed, indicating the importance of ILs for improved conductivity and facilitated exfoliation. The formation of IL-CNRs can be observed by transmission electron microscopy (TEM) (Fig. 2). The length and width of IL-CNRs were about 40 nm and 5 nm (Fig. 2a–c), respectively. Clear lattice lines can be seen from the high-resolution TEM (HRTEM) image (Fig. 2d), indicating good crystallinity. The lattice spaces are 0.26 nm and 0.38 nm, that are consistent with the (100) and (002) lattice of graphite, respectively.^21^

**Fig. 3:**
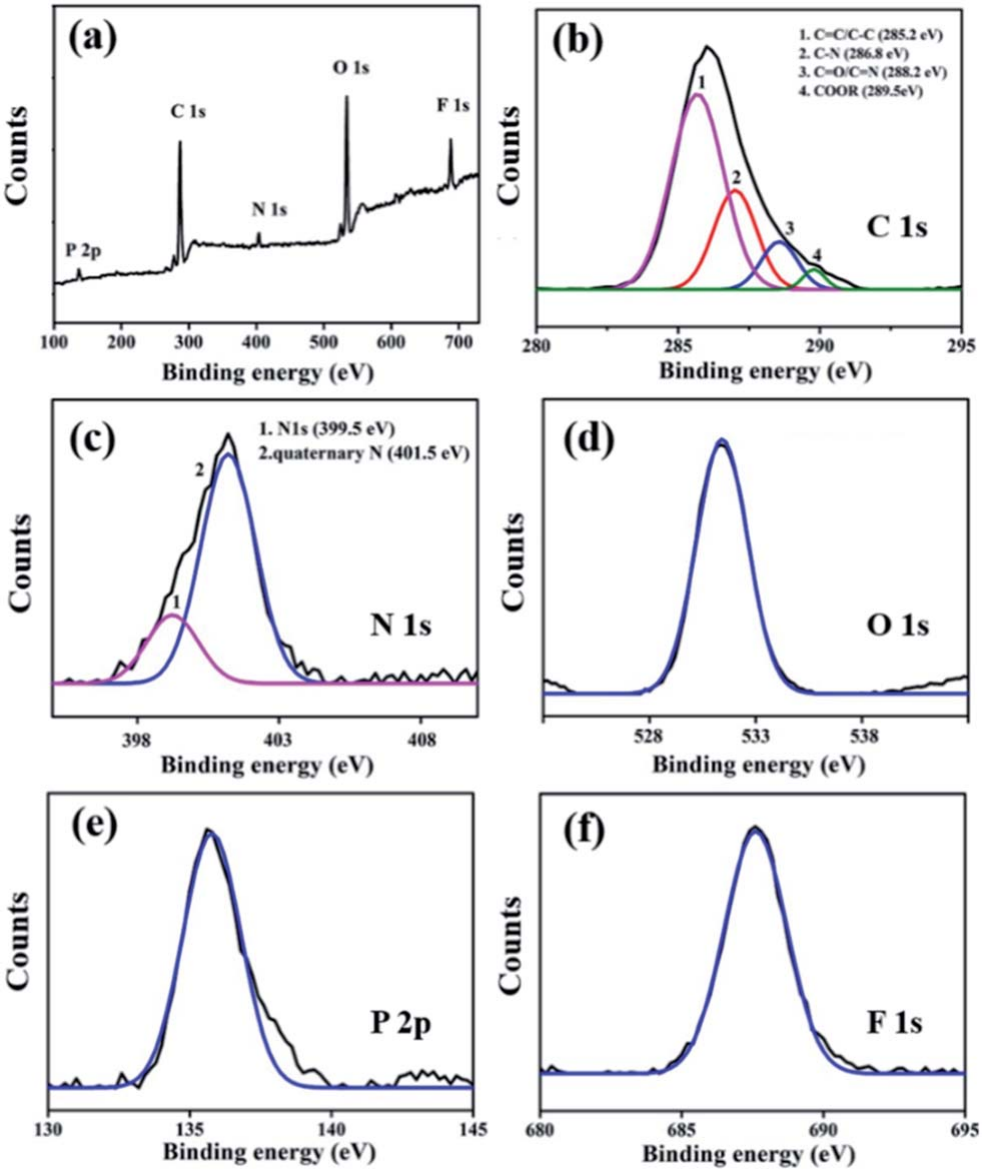
XPS survey spectrum (a) and high resolution C1s (b), N1s (c), O1s (d), P2p (e) and F1s (f) spectra of IL-CNRs.

The chemical composition of the as-prepared IL-CNRs were characterized by X-ray photoelectron spectroscopy (XPS). As shown in Fig. 3a, the XPS survey spectrum of IL-CNRs contains five distinct peaks of C 1s, N 1s, O 1s, P 2p, F 1s. The appearance of N, P, F reveals the formation of IL-CNRs nanocomposites. The C–C/C=C (sp^2^ C) peak is prominent in high resolution spectrum of C 1s, suggesting the graphitic structure in CNRs (Fig. 3b). This result is consistent with the good crystallinity demonstrated in HRTEM image (Fig. 2d). The appearance of C–N in high resolution spectrum of C 1s (Fig. 3b), N–C=C and quaternary N in high resolution spectrum of N 1s (Fig. 3c) confirm the existence of BMIM^+^ in IL-CNRs. The appearance of O 1s reveals the abundant oxygen-containing groups (Fig. 3d), suggesting good solubility of the as-prepared IL-CNRs. The F 1s peak (Fig. 3e) and P 2p peak (Fig. 3f) also prove the presence of PF_6_^−^ anion in IL-CNRs, indicating the hybridization between CNRs and IL. These results evidence the successful formation of [BMIM]PF_6_ modified CNRs in the one-step electrochemical exfoliation of graphite. Consistently, Fourier transform infrared spectrum (FTIR) of IL-CNRs (Fig. S1 in ESI†) also shows characteristic vibrations of C–H (2962, 2922, and 2857 cm^−1^), C=C and C=N (1552 cm^−1^), –OH (3457 cm^−1^), C=O (1640 cm^−1^), and PF_6_^−^ (1578 and 1027 cm^−1^), that agree well with [BMIM]PF_6_. In comparison with IL, UV-Vis spectrum of IL-CNRs exhibits the same large peak of π–π* transition (∼240 nm) and new peak at about 326 nm that corresponds to n–π* transition (Fig. S2 in ESI†). Taken together, [BMIM]PF_6_ are proven to be composited with CNRs.

According to the above results, we propose that the mechanism for the preparation of IL-CNRs includes oxidative cleavage, ionic liquid intercalation and hybridization (Fig. 1).^21,28–31^ Firstly, the oxidation of water is expected to produce radicals (*e.g.* hydroxyl and oxygen radicals) when a high oxidation voltage is applied. Secondly, the oxidation of the edge planes opens up the edge sheets and leads to the expansion of the graphite anode, facilitating intercalation by PF_6_^−^ between the graphene layers. Thirdly, the oxidative cleavage of the expanded graphene sheets generates CNRs. Finally, interaction between CNRs and BMIM^+^*via* π–π or cation–π interactions leads to stable IL-CNRs nanocomposites. In comparison with the preparation of CNRs using electron or ion beam etching, chemical ultrasound, lithographic patterning, oxidative cleavage, and chemical vapor deposition (CVD),^37–40^ this electrochemical method is simple, green and possesses potential for scalable production owing to inexpensive apparatus and easy operation.

### 3.2 Fluorescence properties of IL-CNRs

**Fig. 4:**
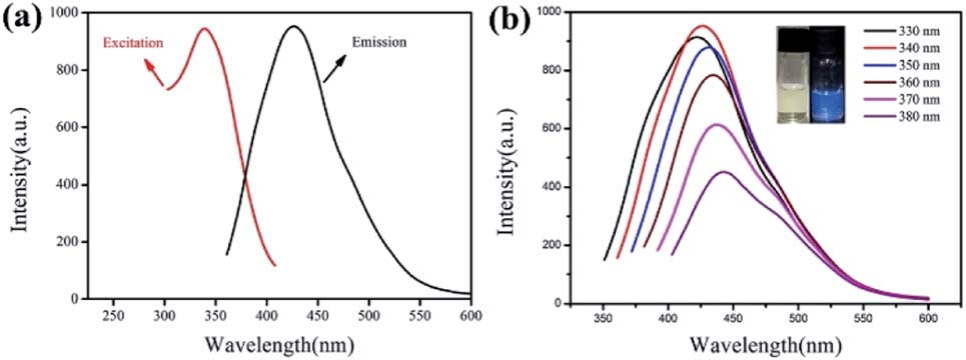
(a) The FL excitation spectrum of IL-CNRs obtained at the emission wavelength of 430 nm and FL emission spectrum of IL-CNRs obtained with the excitation wavelength of 340 nm. (b) The FL emission spectra of IL-CNRs obtained with different excitation wavelengths. Insets are the IL-CNRs solutions under sunlight (left) and UV light (365 nm).

The fluorescence properties of IL-CNRs were investigated and results were demonstrated in Fig. 4. The IL-CNRs show good solubility in water and appears light-yellow under daylight, while exhibit strong blue luminescence under UV light (365 nm) (inset in Fig. 4b). The maximum emission is located at 430 nm and the maximum excitation wavelength is 340 nm (Fig. 4a). Like most luminescent carbon nanomaterials, the IL-CNRs also exhibit excitation-dependent fluorescence behavior (Fig. 4b). IL-CNRs prepared under the same conditions independently exhibit the same maximus emission wavelength and a relative standard deviation (RSD) of 2.9% in fluorescence intensity (at 430 nm), indicating high stability and repeatability of the preparation. As the excitation wavelength increases, the fluorescence emission slightly red-shifts, which could be ascribed to size heterogeneity and distribution of different emissive sites on the carbon ribbons. Absolute photoluminescence quantum yield of IL-CNRs is measured to be 11.4%, which may be ascribed to the high crystallinity of IL-CNRs and the presence of BMIM^+^ on IL-CNRs surface which has electron-withdrawing nitrogen groups. The lifetime of B-GQDs is 5.9 ns (Fig. S3 in ESI†). The IL-CNRs also exhibits good photostability. As shown in Fig. S4 (ESI†), even after being illuminated for 4 h under UV light (365 nm), the fluorescent intensity remains 91%, indicating good anti-photobleaching property.

### 3.3 Ion selectivity of IL-CNRs

**Fig. 5:**
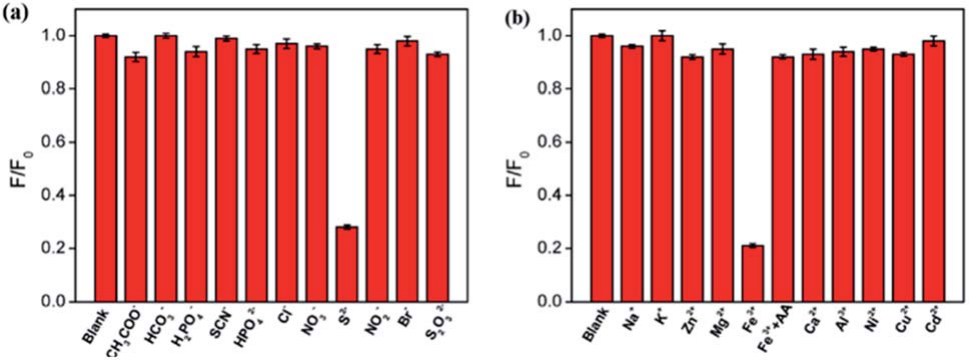
The relative fluorescent ratio of IL-CNRs in presence of different anion (a) or cation (b). The concentration of ions was 0.3 mM.

Ionic liquids modified carbon materials have proven to exhibit anion exchange ability and have potential in direct detection of anion. Thus, the selectivity of the as-prepared IL-CNRs towards different anions were investigated. As revealed in Fig. 5a, the fluorescence of IL-CNRs is strongly quenched by S^2−^, but not the other physiologically or environmentally relevant anions (Fig. 5a). The lifetime of IL-CNRs in presence S^2−^ is also revealed to be 5.9 ns (Fig. S5 in ESI†). The unchanged fluorescence lifetime of IL-CNRs in the presence of S^2−^ indicates no charge transfer and exciton recombination process.^18^ The quenching might be ascribed to the ability of anion exchange originated from IL.^32,33,41^ As fluorescent carbon materials are usually easy to interact with metal cations and cause fluorescence quenching, the selectivity of IL-CNRs towards cations were also investigated. As demonstrated in Fig. 5b, Fe^3+^ cause fluorescent quenching, while other common cations did not significantly affect the fluorescence intensity of the IL-CNRs. The interactions between IL-CNRs and Fe^3+^ could be efficiently masked by ascorbic acid (AA). Thus, IL-CNRs have potential for direct and selective detection of S^2−^ ions.

### 3.4 Detection of S^2−^ ions using IL-CNRs as fluorescent probe

The detection of S^2−^ ions using IL-CNRs as fluorescent probe was investigated. To obtain the highest sensitivity, the detection conditions including pH and incubation time were optimized. As illustrated in Fig. S6a (ESI†), S^2−^ ions could quench the fluorescence of IL-CNRs very quickly. As the incubation time goes beyond 60 s, the fluorescence quenching reaches a plateau. Obviously, IL-CNRs possesses great potential for fast detection of S^2−^ ions. Accordingly, the reaction time with IL-CNRs was set as 60 s in the following experiments. In order to avoid protonation of S^2−^ ions, fluorescence quenching by S^2−^ ions was investigated in near neutral solutions. The highest fluorescent quenching is observed at pH 7, which is chosen for further investigated (Fig. S6b in ESI†).

**Fig. 6:**
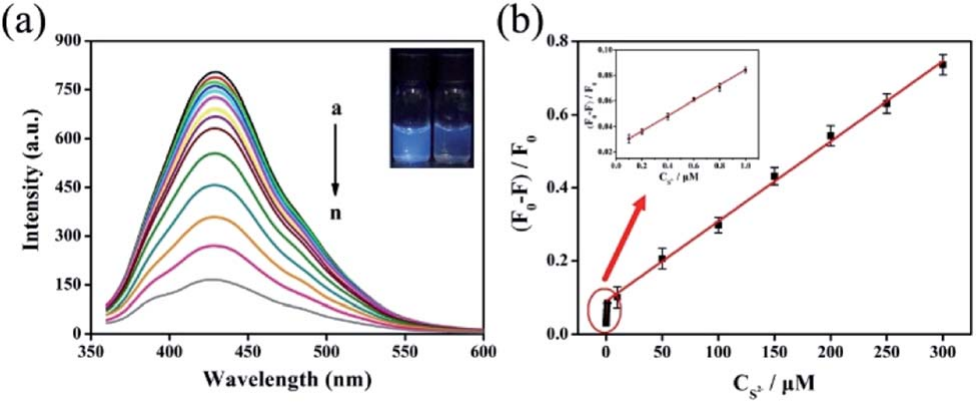
(a) Fluorescence emission spectra of the IL-CNRs upon addition of various concentrations of S^2−^ (from *a* to *n*: 0, 0.1, 0.2, 0.4, 0.6, 0.8, 1, 10, 50, 100, 150, 200, 250, 300 μM). Insets are the digital photos of IL-CNRs in the absence (left) and presence (right) of S^2−^ (100 μM) under UV light (365 nm). (b) The linear dependence of the fluorescence quenching ratio (*F*_0_ − *F*)/*F*_0_ and the concentration of S^2−^ ions. *F*_0_ and *F* were the fluorescence intensity of IL-CNRs in the absence and presence of S^2−^ ions, respectively.

Owing to the fast and sensitive fluorescence quenching of IL-CNRs by S^2−^ ions (insets in Fig. 6a), the fluorescent detection was performed under the optimum conditions. As demonstrated in Fig. 6a, S^2−^ ions caused fluorescence quenching in dose dependent manner. Good linear correlation was found between (*F*_0_ − *F*)/*F*_0_ and the concentration of S^2−^ ions from 100 nM to 1 μM and 1 to 300 μM (Fig. 6b). The limit of detection (LOD) for S^2−^ ions is as low as 85 nM at a signal-to-noise ratio of 3. Thus, IL-CNRs based method is sensitive enough to detect the maximum allowable level of S^2−^ ions in drinking water recommended by World Health Organization.^42,43^ As shown in Table S1 (ESI†), the detection limit obtained with the present method was lower than those obtained by carbon dots (CDs)-Ag^+^,^44^ covalent linking fluorescein isothiocyanate with branched-polyethylenimine (PEI-FITC),^43^ triarylimidazole chromophore (TPI-H)-Cu^2+^,^45^ Au nanoclusters,^46^ lysozyme-stabilized silver nanoclusters (Lys-Ag NCs),^47^ graphene quantum dot (GQDs)-Cu^2+^,^48^ but higher than that obtained using Cu nanoclusters,^49,50^ Au nanoclusters-Ce(iii),^51^ and silver nanoparticles capped with carbon dots (AgNPs-CDs).^52^

### 3.5 Real sample analysis

In order to demonstrate the practical application of the developed fluorescent sensor in detection of S^2−^ in real samples, tap water samples were analyzed by standard addition method. The recoveries ranges from 93.7–103.8% and the relative standard deviations (RSD) is less than 3.0% (Table S2†), indicating the potential for the detection of S^2−^ in complicated real samples.

## 4. Conclusions

Ionic liquid-functionalized carbon nanoribbons (IL-CNRs) were readily synthesized using one-step electrochemical exfoliation of graphite rod in water-IL biphasic system. The hybridization of IL with CNRs not only results in bright fluorescence of CNRs but also endows IL-CNRs with specific interaction with S^2−^ ions. Taking the advantages of bright photoluminescence and selectivity towards S^2−^, IL-CNRs are applied for fast, selective and sensitive detection of S^2−^ ion. In contrast with fluorescent detection of S^2−^ ion using turn-off–on mode, this IL-CNRs demonstrate its potential for practical use in direct detection of anions.

## Conflicts of interest

There are no conflicts to declare.

## Supplementary Material

RA-009-C9RA07701D-s001

## References

[cit1] Karuk Elmas S. N., Ozen F., Koran K., Gorgulu A. O., Sadi G., Yilmaz I., Erdemir S. (2018). Selective and sensitive fluorescent and colorimetric chemosensor for detection of CO_3_^2−^ anions in aqueous solution and living cells. Talanta.

[cit2] Jung J. M., Kang J. H., Han J., Lee H., Lim M. H., Kim K. T., Kim C. (2018). A novel “off–on” type fluorescent chemosensor for detection of Zn^2+^ and its zinc complex for “on–off” fluorescent sensing of sulfide in aqueous solution, in vitro and in vivo. Sens. Actuators, B.

[cit3] Sammi H., Kukkar D., Singh J., Kukkar P., Kaur R., Kaur H., Rawat M., Singh G., Kim K.-H. (2018). Serendipity in solution–GQDs zeolitic imidazole frameworks nanocomposites for highly sensitive detection of sulfide ions. Sens. Actuators, B.

[cit4] Sun Q., Zhang W., Qian J. (2017). A ratiometric fluorescence probe for selective detection of sulfite and its application in realistic samples. Talanta.

[cit5] Yao Y., Sun Q., Chen Z., Huang R., Zhang W., Qian J. (2018). A mitochondria-targeted near infrared ratiometric fluorescent probe for the detection of sulfite in aqueous and in living cells. Talanta.

[cit6] Wu H., Tong C. (2019). Nitrogen- and Sulfur-codoped carbon dots for highly selective and sensitive fluorescent detection of Hg(2+) ions and sulfide in environmental water samples. J. Agric. Food Chem..

[cit7] Wang X., Yang X., Wang N., Lv J., Wang H., Choi M., Bian W. (2018). Graphitic carbon nitride quantum dots as an “off–on” fluorescent switch for determination of mercury(ii) and sulfide. Microchim. Acta.

[cit8] Wang C., Sun J., Mei H., Gao F. (2016). Organic semiconductor polymer nanodots as a new kind of off–on fluorescent probe for sulfide. Microchim. Acta.

[cit9] Sinduja B., John S. A. (2019). Silver nanoparticles capped with carbon dots as a fluorescent probe for the highly sensitive “off–on” sensing of sulfide ions in water. Anal. Bioanal. Chem..

[cit10] Shahbazi N., Zare-Dorabei R. (2019). A novel “off–on” fluorescence nanosensor for sensitive determination of sulfide ions based on carbon quantum dots and gold nanoparticles: Central composite design optimization. Microchem. J..

[cit11] Ramachandran M., Anandan S., Ashokkumar M. (2019). A luminescent on–off probe based calix[4]arene linked through triazole with ruthenium(ii) polypyridine complexes to sense copper(ii) and sulfide ions. New J. Chem..

[cit12] Na W., Qu Z., Chen X., Su X. (2018). A turn-on fluorescent probe for sensitive detection of sulfide anions and ascorbic acid by using sulfanilic acid and glutathione functionalized graphene quantum dots. Sens. Actuators, B.

[cit13] Liu J., Liu C., Zhou Z. (2019). A turn-on fluorescent sulfide probe prepared from carbon dots and MnO_2_ nanosheets. Microchim. Acta.

[cit14] Chen X., Yu S., Yang L., Wang J., Jiang C. (2016). Fluorescence and visual detection of fluoride ions using a photoluminescent graphene oxide paper sensor. Nanoscale.

[cit15] Wang Y., Zhang C., Chen X., Yang B., Yang L., Jiang C., Zhang Z. (2016). Ratiometric fluorescent paper sensor utilizing hybrid carbon dots-quantum dots for the visual determination of copper ions. Nanoscale.

[cit16] Wang H., Yang L., Chu S., Liu B., Zhang Q., Zou L., Yu S., Jiang C. (2019). Semiquantitative visual detection of lead ions with a smartphone via a colorimetric paper-based analytical
device. Anal. Chem..

[cit17] Jiang C., Liu B., Han M., Zhang Z. (2018). Fluorescent nanomaterials for color-multiplexing test papers toward qualitative/quantitative assays. Small Methods.

[cit18] Ge S., He J., Ma C., Liu J., Xi F., Dong X. (2019). One-step synthesis of boron-doped graphene quantum dots for fluorescent sensors and biosensor. Talanta.

[cit19] Lu L., Zhou L., Chen J., Yan F., Liu J., Dong X., Xi F., Chen P. (2018). Nanochannel-confined graphene quantum dots for ultrasensitive electrochemical analysis of complex samples. ACS Nano.

[cit20] Xi F., Zhao J., Shen C., He J., Chen J., Yan Y., Li K., Liu J., Chen P. (2019). Amphiphilic graphene quantum dots as a new class of surfactants. Carbon.

[cit21] Chen B. C., Lin H. P., Chao M. C., Mou C. Y., Tang C. Y. (2004). Mesoporous silica platelets with perpendicular nanochannels via a ternary surfactant system. Adv. Mater..

[cit22] Arjmand M., Sadeghi S., Khajehpour M., Sundararaj U. (2016). Carbon nanotube/graphene nanoribbon/polyvinylidene fluoride hybrid nanocomposites: rheological and dielectric properties. J. Phys. Chem. C.

[cit23] Yan Y., Gong J., Chen J., Zeng Z., Huang W., Pu K., Liu J., Chen P. (2019). Recent advances on graphene quantum dots: from chemistry and physics to applications. Adv. Mater..

[cit24] Wang L., Wang Y., Xu T., Liao H., Yao C., Liu Y., Li Z., Chen Z., Pan D., Sun L., Wu M. (2014). Gram-scale synthesis of single-crystalline graphene quantum dots with superior optical properties. Nat. Commun..

[cit25] Yang Y., Zhou J., Zhang H., Gai P., Zhang X., Chen J. (2013). Electrochemical evaluation of total antioxidant capacities in fruit juice based on the guanine/graphene nanoribbon/glassy carbon electrode. Talanta.

[cit26] Fukumori M., Pandey R. R., Fujiwara T., TermehYousefi A., Negishi R., Kobayashi Y., Tanaka H., Ogawa T. (2017). Diameter dependence of longitudinal unzipping of single-walled carbon nanotube to obtain graphene nanoribbon. Jpn. J. Appl. Phys..

[cit27] Sadeghi S., Arjmand M., Otero Navas I., Zehtab Yazdi A., Sundararaj U. (2017). Effect of nanofiller geometry on network formation in polymeric nanocomposites: comparison of rheological and electrical properties of multiwalled carbon nanotube and graphene nanoribbon. Macromolecules.

[cit28] Ananthanarayanan A., Wang X., Routh P., Sana B., Lim S., Kim D.-H., Lim K.-H., Li J., Chen P. (2014). Facile synthesis of graphene quantum dots from 3D graphene and their application for Fe^3+^ sensing. Adv. Funct. Mater..

[cit29] Xu Y., Liu J., Zhang J., Zong X., Jia X., Li D., Wang E. (2015). Chip-based generation of carbon nanodots via electrochemical oxidation of screen printed carbon electrodes and the applications for efficient cell imaging and electrochemiluminescence enhancement. Nanoscale.

[cit30] Li H., Chen L., Wu H., He H., Jin Y. (2014). Ionic liquid-functionalized fluorescent carbon nanodots and their applications in electrocatalysis, biosensing, and cell imaging. Langmuir.

[cit31] Li X., Zhao Z. (2014). Facile ionic-liquid-assisted electrochemical synthesis of size-controlled carbon quantum dots by tuning applied voltages. RSC Adv..

[cit32] Wang B., Tang W., Lu H., Huang Z. (2015). Hydrothermal synthesis of ionic liquid-capped carbon quantum dots with high thermal stability and anion responsiveness. J. Mater. Sci..

[cit33] Sun X., Qian Y., Jiao Y., Liu J., Xi F., Dong X. (2017). Ionic liquid-capped graphene quantum dots as label-free fluorescent probe for direct detection of ferricyanide. Talanta.

[cit34] Ali M. E. A. (2019). Preparation of graphene nanosheets by electrochemical exfoliation of a graphite-nanoclay composite electrode: application for the adsorption of organic dyes. Colloids Surf., A.

[cit35] Chen D., Wang F., Li Y., Wang W. W., Huang T. X., Li J. F., Novoselov K. S., Tian Z. Q., Zhan D. (2019). Programmed electrochemical exfoliation of graphite to high quality graphene. Chem. Commun..

[cit36] Fu Y., Gao G., Zhi J. (2019). Electrochemical synthesis of multicolor fluorescent N-doped graphene quantum dots as a ferric ion sensor and their application in bioimaging. J. Mater. Chem. B.

[cit37] Li X., Wang X., Zhang L., Lee S., Dai H. (2008). Chemically derived, ultrasmooth graphene nanoribbon semiconductors. Science.

[cit38] Jin C., Lan H., Peng L., Suenaga K., Iijima S. (2009). Deriving carbon atomic chains from graphene. Phys. Rev. Lett..

[cit39] Tapaszto L., Dobrik G., Lambin P., Biro L. P. (2008). Tailoring the atomic structure of graphene nanoribbons by scanning tunnelling microscope lithography. Nat. Nanotechnol..

[cit40] Lemme M. C., Bell D. C., Williams J. R., Stern L. A., Baugher B. W. H., Jarillo-Herrero P., Marcus C. M. (2009). Etching of graphene devices with a helium ion beam. ACS Nano.

[cit41] Wang B., Song A., Feng L., Ruan H., Li H., Dong S., Hao J. (2015). Tunable amphiphilicity and multifunctional applications of ionic liquid-modified carbon quantum dots. ACS Appl. Mater. Interfaces.

[cit42] Chen J., Li Y., Lv K., Zhong W., Wang H., Wu Z., Yi P., Jiang J. (2016). Cyclam-functionalized carbon dots sensor for sensitive and selective detection of copper(ii) ion and sulfide anion in aqueous media and its imaging in live cells. Sens. Actuators, B.

[cit43] Lv K., Chen J., Wang H., Zhang P., Yu M., Long Y., Yi P. (2017). One-pot fabrication of FRET-based fluorescent probe for detecting copper ion and sulfide anion in 100% aqueous media. Spectrochim. Acta, Part A.

[cit44] Barati A., Shamsipur M., Abdollahi H. (2016). Metal-ion-mediated fluorescent carbon dots for indirect detection of sulfide ions. Sens. Actuators, B.

[cit45] Wang Y., Qiu D., Li M., Liu Y., Chen H., Li H. (2017). A new “on–off–on” fluorescent probe containing triarylimidazole chromophore to sequentially detect copper and sulfide ions. Spectrochim. Acta, Part A.

[cit46] Wang L., Chen G., Zeng G., Liang J., Dong H., Yan M., Li Z., Guo Z., Tao W., Peng L. (2015). Fluorescent sensing of sulfide ions
based on papain-directed gold nanoclusters. New J. Chem..

[cit47] Sun H., Lu D., Xian M., Dong C., Shuang S. (2016). A lysozyme-stabilized silver nanocluster fluorescent probe for the detection of sulfide ions. Anal. Methods.

[cit48] Yu N., Peng H., Xiong H., Wu X., Wang X., Li Y., Chen L. (2015). Graphene quantum dots combined with copper(ii) ions as a fluorescent probe for turn-on detection of sulfide ions. Microchim. Acta.

[cit49] Chen J., Li Y., Zhong W., Hou Q., Wang H., Sun X., Yi P., Jiang J. (2015). Novel fluorescent polymeric nanoparticles for highly selective recognition of copper ion and sulfide anion in water. Sens. Actuators, B.

[cit50] Li Z., Guo S., Lu C. (2015). A highly selective fluorescent probe for sulphide ions based on aggregation of Cu nanocluster induced emission enhancement. Analyst.

[cit51] Liu J., Bao H., Ma D., Leung C. (2019). Silver nanoclusters functionalized with Ce(iii) ions are a viable “turn-on–off” fluorescent probe for sulphide. Microchim. Acta.

[cit52] Sinduja B., Abraham John S. (2019). Silver nanoparticles capped with carbon dots as a fluorescent probe for the highly sensitive “off–on” sensing of sulfide ions in water. Anal. Bioanal. Chem..

