# Supplementary material for: Direct and sensitive detection of sulfide ions based on one-step synthesis of ionic liquid functionalized fluorescent carbon nanoribbons
Source: RSC Adv. 2019 Nov 15;9(64):37484–90. doi: 10.1039/c9ra07701d (PMC9075588; doi:10.1039/c9ra07701d)
Supplement: RA-009-C9RA07701D-s001 [file RA-009-C9RA07701D-s001.pdf]

Electronic Supplementary Material (ESI) for RSC Advances.
This journal is © The Royal Society of Chemistry 2019

Supplementary Material:

Tables

Table S1. Comparison between fluorescent detection of S^{2-} using different probes.

Fluorescent probe	DL (μM)	Linear range (μM)	Ref.
PEI-FITC-Cu^{2+}	1.2	10-100	43
CDs-Ag^{+}	0.43	0-100	44
TPI-H-Cu^{2+}	0.156	-	45
Au nanoclusters	0.38	0.5-80	46
Lys-Ag NCs	1.1	5-100	47
GQDs-Cu^{2+}	0.1	0.2-20	48
Cu nanoclusters	0.01	0.02–0.8	49
Cu nanoclusters	0.042	0.2–50	50
Au nanoclusters-Ce(III)	0.015	0-2	51
AgNPs-CDs	0.01	0.01-0.9	52
IL-CNRs	0.085	0.1-1.0, 1.0-300	This work

CDs-Ag^{+}, carbon dots-Ag^{+}; PEI-FITC, covalent linking fluorescein isothiocyanate with branched-polyethylenimine; TPI-H, triarylimidazole chromophore; Lys-Ag NCs, lysozyme-stabilized silver nanoclusters; Eu^{3+}-BHHCT-BPED, AgNPs-CDs, silver nanoparticles capped with carbon dots; GQDs, graphene quantum dots.

Table S2. Detection of S^{2-} in tap water samples.

Sample[a]	Concentration of S^{2-} (μM)		RSD (n=3, %)	Recovery (%)
	Added	Determined by IL-CNRs		
1	0.80	0.83	2.3	103.8
2	10.00	9.37	2.5	93.7
3	50.00	48.44	1.9	96.9

[a] S^{2-} in the original tap water is not detected. Recovery=determined concentration/added value×100%.

Figures

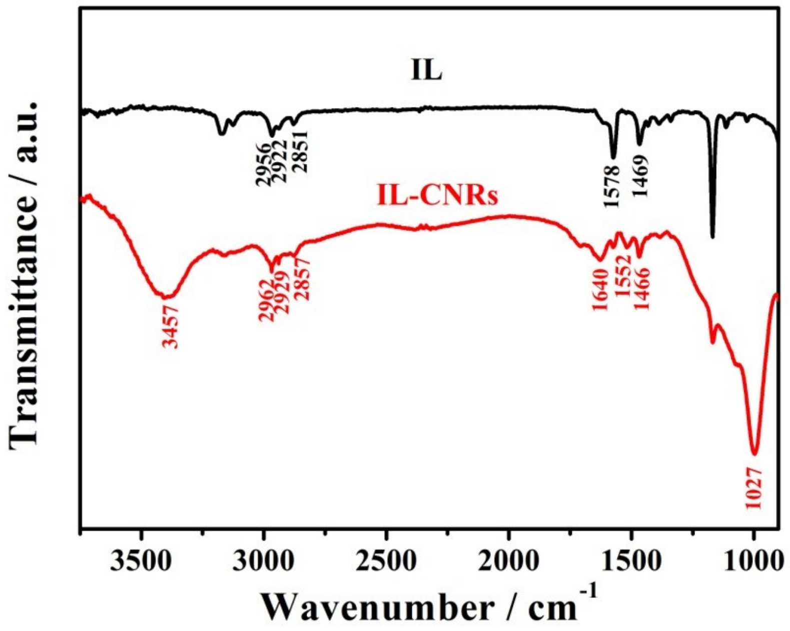

Fig. S1. FTIR spectra of IL and IL-CNRs.

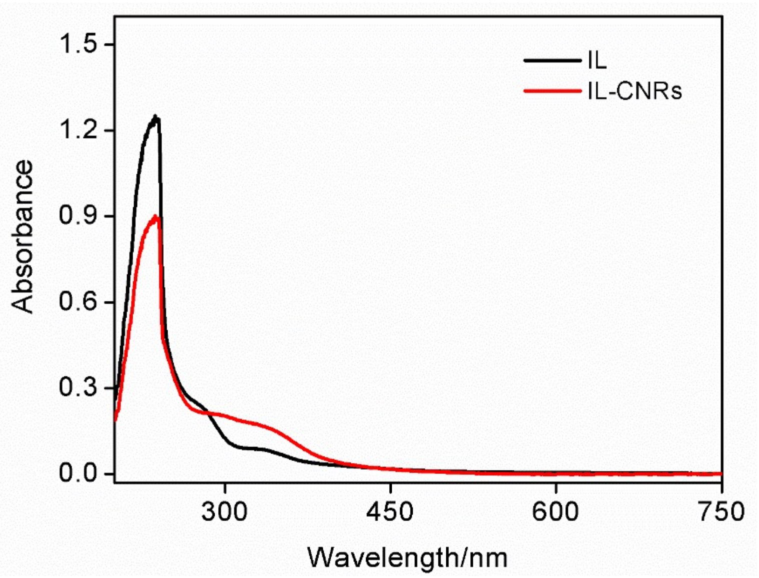

Fig. S2. UV-Vis spectra of IL and IL-CNRs.

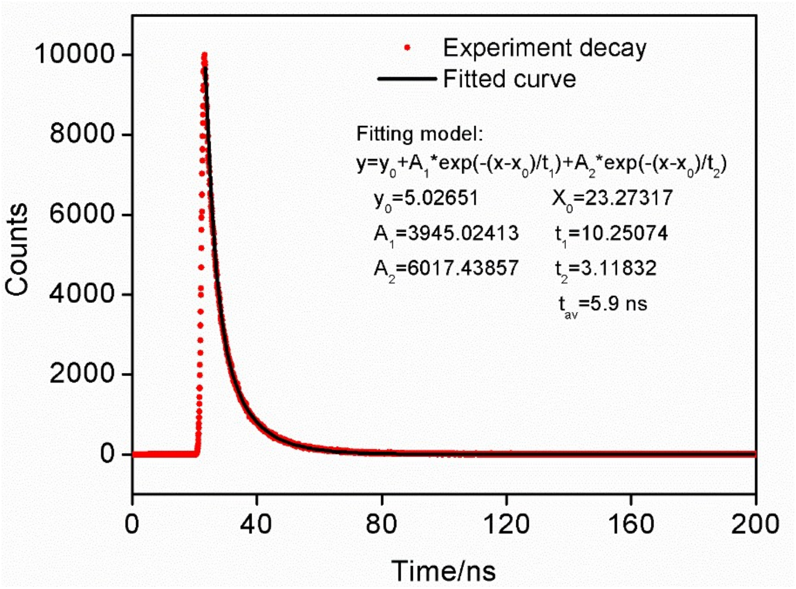

Fig. S3. Fluorescence lifetime spectrum of IL-CNRs.

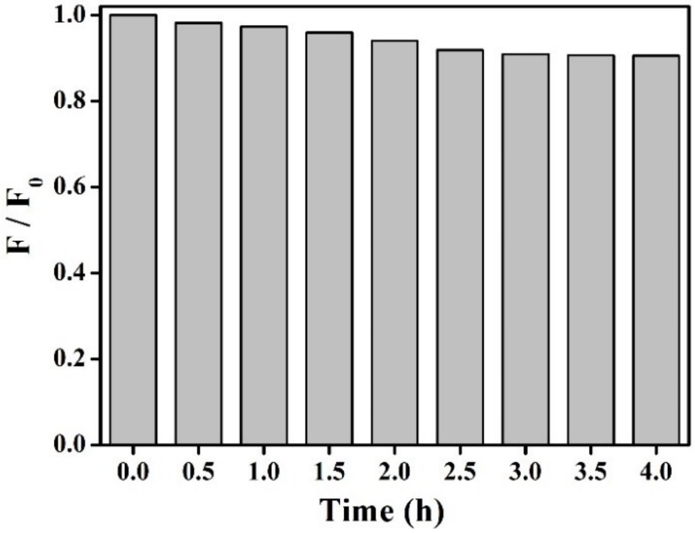

Fig. S4. The fluorescence intensity ratio of IL-CNRs under UV light (365 nm) irradiation for different time. F_0 and F were the fluorescence intensity of IL-CNRs before and after UV light irradiation, respectively.

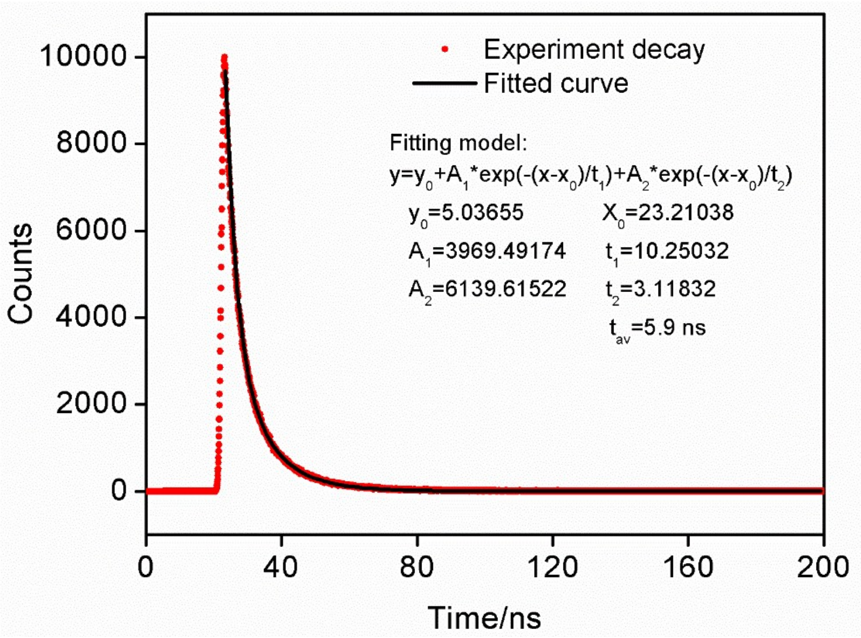

Fig. S5. Fluorescence lifetime spectrum of IL-CNRs in presence of S^{2-}.

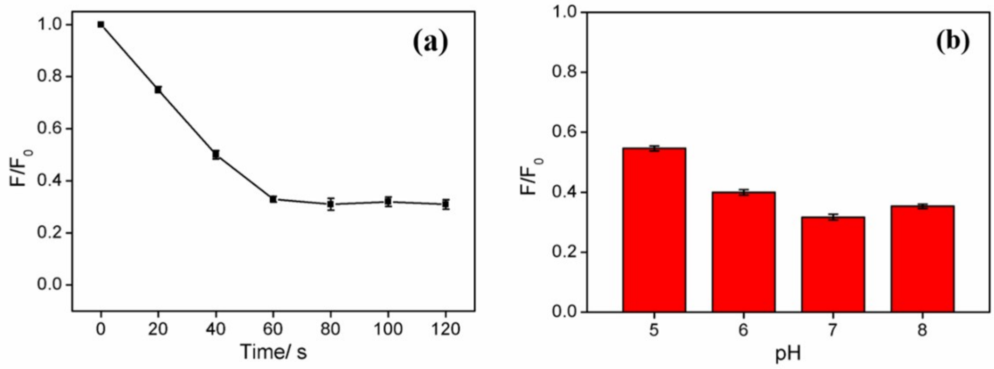

Fig. S6. The effects of incubation time and pH on the fluorescence intensity ratio by S^{2-}.
